# A screening method for mild cognitive impairment in elderly individuals combining bioimpedance and MMSE

**DOI:** 10.3389/fnagi.2024.1307204

**Published:** 2024-01-24

**Authors:** Min-Ho Jun, Boncho Ku, Kahye Kim, Kun Ho Lee, Jaeuk U. Kim

**Affiliations:** ^1^Digital Health Research Division, Korea Institute of Oriental Medicine (KIOM), Daejeon, Republic of Korea; ^2^School of Korean Convergence Medical Science, University of Science and Technology, Daejeon, Republic of Korea; ^3^Gwangju Alzheimer’s Disease and Related Dementias (GARD) Cohort Research Center, Chosun University, Gwangju, Republic of Korea; ^4^Department of Biomedical Science, Chosun University, Gwangju, Republic of Korea; ^5^Dementia Research Group, Korea Brain Research Institute, Daegu, Republic of Korea

**Keywords:** bioimpedance analysis, mild cognitive impairment, dementia, screening, combination of bioimpedance and MMSE

## Abstract

We investigated a screening method for mild cognitive impairment (MCI) that combined bioimpedance features and the Korean Mini-Mental State Examination (K-MMSE) score. Data were collected from 539 subjects aged 60 years or older at the Gwangju Alzheimer’s & Related Dementias (GARD) Cohort Research Center, A total of 470 participants were used for the analysis, including 318 normal controls and 152 MCI participants. We measured bioimpedance, K-MMSE, and the Seoul Neuropsychological Screening Battery (SNSB-II). We developed a multiple linear regression model to predict MCI by combining bioimpedance variables and K-MMSE total score and compared the model’s accuracy with SNSB-II domain scores by the area under the receiver operating characteristic curve (AUROC). We additionally compared the model performance with several machine learning models such as extreme gradient boosting, random forest, support vector machine, and elastic net. To test the model performances, the dataset was divided into a training set (70%) and a test set (30%). The AUROC values of SNSB-II scores were 0.803 in both sexes, 0.840 for males, and 0.770 for females. In the combined model, the AUROC values were 0.790 (0.773) for males (and females), which were significantly higher than those from the model including MMSE scores alone (0.723 for males and 0.622 for females) or bioimpedance variables alone (0.640 for males and 0.615 for females). Furthermore, the accuracies of the combined model were comparable to those of machine learning models. The bioimpedance-MMSE combined model effectively distinguished the MCI participants and suggests a technique for rapid and improved screening of the elderly population at risk of cognitive impairment.

## 1 Introduction

Population aging is a global phenomenon because of improvement in hygiene, nutritional status, and medical technology. Consequently, the number of elderly individuals at risk of dementia or mild cognitive impairment (MCI) is increasing at an alarming rate. Dementia imposes a substantial social burden due to its challenging treatment and the suffering experienced by patients and their care givers. Early detection of cognitive impairment may help to prevent or slow the progression of dementia.

To identify cognitive dysfunction, clinics often employ neuropsychological assessment questionnaires, such as the Consortium to Establish a Registry for Alzheimer’s Disease (CERAD), Dementia Rating Scale (DRS), Alzheimer’s Disease Assessment Scale-Cognitive Subscale (ADAS-Cog), and Seoul Neuropsychological Screening Battery II (SNSB II). These assessments are widely utilized for the comprehensive cognition test due to their accessibility, cost-effectiveness, convenience, and the use of neuroimaging techniques such as magnetic resonance imaging (MRI) or positron emission tomography (PET) to determine the underlying causes of cognitive impairment (Benedict and Zivadinov, 2011; Risacher et al., 2017; Petracca et al., 2021).

However, these comprehensive neuropsychological assessments may not be readily available to many participants due to their time-consuming nature and the need for a professionally trained practitioner. As a result, brief neuropsychological questionnaires such as the Mini-Mental State Examination (MMSE) and the Montreal Cognitive Assessment (MoCA) have gained widespread popularity as screening tools for dementia (Folstein et al., 1975; Nasreddine et al., 2005; Smith et al., 2007; Roalf et al., 2013). These tools are easily administered with minimal training, and can be completed within 10 min and have demonstrated diagnostic utility. The MMSE, in particular, is the most widely adopted measure for screening cognitive function in medical and neuropsychological research. However, the MMSE is known to lack its accuracy to earlier cognitive impairment from cognitively normal elderly (Hoops et al., 2009; Lee et al., 2009; Arevalo-Rodriguez et al., 2015; Ciesielska et al., 2016).

Bioelectrical impedance analysis (BIA), also known as bioimpedance analysis, is widely acknowledged to be a safe, rapid, reliable, easy-to-use, portable, noninvasive, and cost-effective technique. BIA has extensive applications in measuring body composition (Abu Khaled et al., 1988; Jackson et al., 1988; Shafer et al., 2009). Recently, numerous studies have reported the potential of using BIA measurements to assess health indicators and clinical outcomes among various patient populations. Specifically, BIA has been extensively used as a valuable index for monitoring and screening different diseases and conditions, including mortality, nutrition status, diabetes, hemodialysis, chronic heart failure, and liver cirrhosis (Selberg and Selberg, 2002; Colin-Ramirez et al., 2012; Beberashvili et al., 2014; Dittmar et al., 2015; Genton et al., 2017; Kuchnia et al., 2017; Jun et al., 2018). Some studies have explored the association between cognitive function, such as MCI or dementia related to Alzheimer’s disease (AD), and BIA variables. Notably, BIA variables were significantly different in individuals with AD compared to controls. Participants with AD exhibited lower lean tissue mass and higher percent fat mass than healthy elderly individuals. Reactance and the ratio of reactance to height were approximately 21% lower in individuals with dementia than in older adults without dementia (Buffa et al., 2010, 2014; Camina Martin et al., 2015). Additionally, AD patients demonstrated significantly higher height-normalized impedance values and lower phase angles (indicative of body cell mass) than healthy controls. Men with MCI exhibited a higher ratio of impedance to height than healthy controls (Cova et al., 2017). Furthermore, a study reported positive correlations between muscle mass percentage and cognitive function measures such as attention and executive function (Crespillo-Jurado et al., 2019). Recent research has also revealed associations between MCI and increased segmental water and lean mass in the lower extremities, as well as decreased resistance and reactance in the lower extremities (Doan et al., 2022b).

Numerous studies have highlighted the relationship between cognitive function and body composition, which can be influenced by factors such as nutrition status and physical activity. However, a screening method for cognitive in a manner that is easy, cost-effective, rapid, and relatively accurate is challenging. In this study, we propose a new approach to screen for MCI by combining MMSE with BIA measurements.

To evaluate the classification accuracy of prediction models that distinguish individuals with MCI from cognitively normal controls (NCs), we plotted a receiver operating characteristic (ROC) curve for the combined model (including MMSE scores and BIA measurements) and calculated the corresponding area under the ROC (AUROC). Specifically, we compared the AUROC value of the model combining the MMSE scores and BIA measures with that of a model with SNSB II scores. Furthermore, we explored the possibility of diagnosing MCI using various machine learning algorithms, by comparing their performance via AUROC values.

The results of this study have the potential to facilitate easy, rapid, and relatively accurate screening of individuals suspected to have cognitive impairment within a large population. It can help identify individuals who need for further precision neuropsychological tests and/or neuroimaging in clinical settings, thereby saving energy and medical cost.

## 2 Materials and methods

### 2.1 Participants

From December 2017 to February 2020, participants were recruited from the GARD Cohort Research Center for the early diagnosis and progression of Alzheimer’s disease for local citizens aged 60 years or older from Gwangju city, South Korea. A total of 539 participants were involved in this study, and data from 470 participants were used for the analysis, excluding 69 individuals with no data, failure to meet the criteria, or deferred diagnosis. There were 152 individuals with clinical consensus regarding a diagnosis of MCI and 318 elderly normal individuals, for a total of 470 participants. The sex ratio of the analyzed sample consisted of 219 males and 251 females. The study was approved by the Institutional Review Board (IRB) of Chonnam National University Hospital and Chosun University Hospital (IRB number: CNUH-2019-279, CHOSUN 2016–10–005-017). All participants were informed of the objectives and methods of the research, and they provided written informed consent. The study was performed according to the Declaration of Helsinki guidelines.

### 2.2 Neuropsychological assessment

The Korean version of MMSE (K-MMSE) and the SNSB-II were used for neuropsychological evaluations. The K-MMSE incorporates a range of cognitive domains that include orientation in time (5 points), orientation in place (5 points), memory registration (3 points), attention and calculation (5 points), memory recall (3 points), language (8 points), and spatiotemporal configuration (1 point) (Kang et al., 2016). This creates a total score of 30 points. The SNSB-II assesses attention, language, memory, visuospatial skills, and frontal/executive function; The memory domain includes the Seoul Verbal Learning Test (SVLT) and the Rey Complex Figure Test (RCFT) (Jahng et al., 2015). The frontal/executive function domain includes motor impersistence, contrasting program, the go-no-go task, the Luria test, alternating hand movements, the alternating square and triangle task, the Luria loop task, Controlled Oral Word Association Test (COWAT), Korean-Color Word Stroop Test (K-CWST), Digit Symbol Coding (DSC), and Korean-Trail Making Test. The language domain includes the Korean-Boston Naming Test (K-BNT), right–left orientation, and calculation. The attention domain includes the Digit Span Test (DST), letter cancellation, and vigilance test. The visuospatial domain is composed of the clock drawing test (CDT) and RCFT. It also includes other related tests, such as the Clinical Dementia Rating Scale (CDR), Barthel-Activities of Daily Living (B-ADL), Korean-Instrumental Activities of Daily Living (K-IADL), and Geriatric Depression Scale (GDS). The estimated completion time of the whole battery is one and a half hours to two hours.

The diagnostic criteria for MCI proposed by the International Working Group on MCI were the absence of dementia according to the criteria of the Diagnostic and Statistical Manual of Mental Disorders, fifth edition (DSM-V) (Edition F, 2013). Detailly, we evaluated all participants using comprehensive clinical consultations involving a combination of a neuropsychological battery and the Clinical Dementia Rating (CDR) scale. Individuals categorized as cognitively normal (CN) were clinically identified with a CDR score of zero and displayed no cognitive impairment. Meanwhile, those with a CDR score of 0.5 and evidence of cognitive decline in one or more domains, were classified as having MCI. MCI patients exhibited a Seoul Neuropsychological Screening Battery-Second Edition (SNSB-II) z score of less than −1.5 in at least one cognitive domain.

### 2.3 Bioimpedance analysis measurements

Bioelectrical impedance was measured using a direct segmental multifrequency bioelectrical impedance analyzer with tetrapolar 8-point electrodes (InBody S10, InBody, Korea). We measured the impedance at six frequencies (1, 5, 50, 250, 500, and 1,000 kHz) and reactance and phase angle (PhA) values at three frequencies (5, 50, and 250 kHz). Eight electrodes were used to measure segmental impedance in five body segments (the two arms, two legs, and trunk). After the readings sufficiently stabilized, BIA measurements were collected from the limbs of the body with the subjects in a supine position, which is one of the standard measurement positions.

### 2.4 Data selection and statistical analysis

All statistical analyses were performed with R (version 4.0.3, released on 2020-10-10) (Team RC, 2019). Individuals with invalid data (69 participants) or AD dementia (three participants) were excluded. An independent-sample *t*-test was carried out to assess the differences in means of variables between participants with MCI and healthy controls. The correlations between BIA variables and domain scores and total scores on the neuropsychological questionnaires were analyzed. A prediction model for MCI was constructed by combining BIA variables and the K-MMSE total score, and AUROC values of the combined model were compared to those of the model with the SNSB-II scores. Although the data were not extensive, the classification accuracy of several machine learning algorithms (constructed in R) was calculated in terms of AUROC values, and the differences between the prediction models with various variables and the variables included by the machine learning algorithms were compared.

To verify the models developed in this study, we conducted machine learning experiments with several well-known machine learning algorithms on different feature sets. The candidate feature sets consisted of the following:

1. Demographic characteristics (DM), including age, sex, education level, height, weight, and BMI
2. BIA variables (including raw signals) with effect sizes greater than 0.2 (BIA feat. filtered)
3. DM + MMSE scores
4. DM + MMSE scores + BIA feat. filtered
5. DM + BIA feat. filtered
6. Features identified with a greedy search algorithm and a binary logistic regression model, previously used in the proposed models

All models based on different feature sets in this experiment were evaluated using datasets stratified by sex and the entire dataset. The candidate feature sets were randomly and identically divided into a training set comprising 70% of the data (females: 172, males: 157, total participants: 329) and a test set comprising 30% of the data (females: 79, males: 62, total participants: 141), with a similar distribution of normal individuals and MCI participants as in the original dataset. To compensate for the imbalance of data in the distribution of NC and MCI participants, the synthetic minority oversampling technique (SMOTE) was applied to all candidate feature sets.

All continuous features were transformed using two methods, z-transformation and min-max transformation, to examine the effect of the different normalization methods. We trained four different machine learning (ML) algorithms on each feature set: Elastic-net logistic regression (ELASTIC-NET) (Zou and Hastie, 2005), support vector machine with a radial basis kernel (svmRBF) (Scholkopf et al., 1997), random forest (RF) (Breiman, 2001), and extreme gradient boosting (XGBOOST) (Chen and Guestrin, 2016). The optimal hyperparameters in the training of each candidate feature set were selected via two rounds of 5-fold cross-validation. The performance of the models was evaluated using the AUROC values of the test sets, considering the combination of the sex-stratified datasets, entire dataset, candidate feature sets, candidate models, and normalization methods. All pipelines in this machine learning experiment were implemented using the tidymodels package within R.

## 3 Results

### 3.1 Patient demographic and clinical characteristics

The ages (mean ± SD) of male participants with MCI (*n* = 78) and normal controls (NCs) (*n* = 141) were 73.78 ± 6.35 years and 75.33 ± 6.61 years, respectively, and the ages of female participants with MCI (*n* = 74) and NCs (*n* = 177) were 70.77 ± 6.29 years and 71.43 ± 6.21 years, respectively. The demographic characteristics, family history, medical history, and neuropsychology test data, according to sex and cognitive status, are summarized in Table 1. BIA data are presented in Table 2 according to sex due to the large difference in body composition between males and females. The age, weight, height, body mass index (BMI), and years of education of participants with MCI and healthy controls were not significantly different according to t tests for males or females. Neuropsychological test scores (on the K-MMSE and SNSB-II) showed significant differences between participants with MCI and NCs in both sexes.

**Table 1:** Demographic characteristics according to sex and cognitive status.

	**Male**					**Female**				
	**NC** **(*N* = 141)** ^**1**^	**MCI** **(*N* = 78)** ^**1**^	***D*** ^**2**^	**95% CI** ^**2**^	***P*-value** ^**2**^	**NC** **(*N* = 177)** ^**1**^	**MCI** **(*N* = 74)** ^**1**^	***D*** ^**2**^	**95% CI** ^**2**^	***P*-value** ^**2**^
**Demographics**										
Age (yrs.)	73.78 (6.35)	75.33 (6.61)	−1.56	−3.35, 0.24	0.089	70.77 (6.29)	71.43 (6.21)	−0.66	−2.37, 1.05	0.446
Education (yrs.)	15.09 (4.96)	14.72 (5.03)	0.37	−1.02, 1.75	0.602	11.55 (3.96)	12.11 (4.53)	−0.55	−1.68, 0.57	0.334
Height (cm)	166.47 (5.43)	165.80 (5.39)	0.67	−0.84, 2.17	0.383	153.50 (5.10)	153.29 (6.28)	0.21	−1.28, 1.70	0.783
Weight (kg)	68.21 (8.54)	67.92 (9.07)	0.29	−2.14, 2.71	0.816	58.38 (7.27)	58.46 (10.76)	−0.08	−2.38, 2.22	0.946
BMI (kg/m^2^)	24.58 (2.57)	24.69 (2.92)	−0.11	−0.86, 0.64	0.774	24.79 (3.02)	24.79 (3.82)	0.00	−0.89, 0.90	0.993
K-MMSE	27.91 (1.65)	26.12 (2.44)	1.80	1.25, 2.35	<0.001	27.27 (1.95)	26.03 (2.82)	1.24	0.63, 1.85	<0.001
**Family history**										
Dementia	38 (27%)	17 (22%)	5.2%	−7.6, 18%	0.497	47 (27%)	19 (26%)	0.88%	−12, 14%	>0.999
Stroke	32 (23%)	9 (12%)	11%	0.26, 22%	0.065	43 (24%)	20 (27%)	−2.7%	−16, 10%	0.767
**Medical history**										
Heart diseases^†^	23 (16%)	12 (15%)	0.93%	−10, 12%	>0.999	22 (12%)	15 (20%)	−7.8%	−19, 3.5%	0.161
Hypertension	57 (40%)	38 (49%)	−8.3%	−23, 6.4%	0.297	71 (40%)	27 (36%)	3.6%	−10, 18%	0.693
Diabetes	29 (21%)	27 (35%)	−14%	−28, −0.56%	0.034	31 (18%)	14 (19%)	−1.4%	−13, 10%	0.933
Mental disease^††^	1 (0.7%)	2 (2.6%)	−1.9%	−6.6, 2.9%	0.600	4 (2.3%)	2 (2.7%)	−0.44%	−5.2, 4.3%	>0.999
**SNSB-II domains**										
Attention	10.09 (2.22)	8.54 (1.82)	1.55	0.97, 2.13	<0.001	9.42 (2.05)	8.53 (2.01)	0.90	0.34, 1.45	0.002
Language	0.27 (0.28)	−0.03 (0.50)	0.30	0.19, 0.40	<0.001	0.13 (0.35)	−0.10 (0.44)	0.23	0.13, 0.33	<0.001
Visuospatial	0.58 (0.35)	0.22 (0.82)	0.36	0.20, 0.51	<0.001	0.53 (0.35)	0.12 (0.85)	0.41	0.26, 0.56	<0.001
Memory	0.30 (0.61)	−0.54 (0.61)	0.84	0.67, 1.00	<0.001	0.29 (0.57)	−0.26 (0.60)	0.55	0.39, 0.71	<0.001
Frontal/Executive	0.31 (0.59)	−0.29 (0.66)	0.61	0.44, 0.78	<0.001	0.21 (0.58)	−0.26 (0.68)	0.47	0.30, 0.64	<0.001

^1^Mean (SD); n (%). ^2^Two Sample *t*-test; Two sample test for equality of proportions. ^†^ Including CVD, heart failure, arrhythmia, and etc. ^††^ Including depression, alcohol addiction, and etc. NC, normal control; MCI, mild cognitive impairment; D, mean difference or proportion difference; CI, confidence interval; K-MMSE, Korean mini-mental state examination; SNSB, Seoul Neuropsychological Screening Battery Scores.

**Table 2:** BIA measures according to sex and cognitive status.

	**Male**					**Female**				
	**NC** **(*N* = 141)**^**1**^	**MCI** **(*N* = 78)** ^**1**^	***D*** ^**2**^	**95% CI** ^**2**^	***P*-value** ^**2**^	**NC** **(*N* = 177)** ^**1**^	**MCI** **(*N* = 74)** ^**1**^	***D*** ^**2**^	**95% CI** ^**2**^	***P*-value** ^**2**^
**S-10 features**										
ICW (L)	22.23 (2.53)	21.69 (2.36)	0.54	−0.14, 1.23	0.121	16.21 (1.68)	16.15 (2.03)	0.06	−0.43, 0.55	0.809
ECW (L)	14.22 (1.51)	14.03 (1.50)	0.19	−0.23, 0.61	0.364	10.52 (1.02)	10.59 (1.31)	−0.07	−0.38, 0.23	0.640
TBW (L)	36.45 (4.00)	35.71 (3.81)	0.74	−0.36, 1.83	0.186	26.73 (2.68)	26.74 (3.33)	−0.01	−0.80, 0.77	0.975
FAT (kg)	18.70 (5.39)	19.47 (6.04)	−0.77	−2.34, 0.80	0.333	22.00 (5.56)	21.85 (7.55)	0.15	−1.54, 1.84	0.862
SLM (kg)	46.64 (5.15)	45.67 (4.89)	0.97	−0.44, 2.38	0.177	34.17 (3.44)	34.15 (4.26)	0.02	−0.99, 1.03	0.971
FFM (kg)	49.28 (5.45)	48.26 (5.15)	1.02	−0.47, 2.51	0.178	36.23 (3.62)	36.20 (4.49)	0.03	−1.04, 1.09	0.963
SMM (kg)	26.99 (3.31)	26.29 (3.08)	0.70	−0.19, 1.60	0.124	19.14 (2.19)	19.06 (2.65)	0.07	−0.56, 0.71	0.821
PBF (%)	27.15 (5.83)	28.24 (5.98)	−1.09	−2.72, 0.55	0.192	37.34 (5.82)	36.78 (6.62)	0.56	−1.09, 2.21	0.506
WHR	0.884 (0.057)	0.888 (0.068)	−0.003	−0.020, 0.014	0.703	0.886 (0.051)	0.881 (0.066)	0.005	−0.010, 0.020	0.479
ECW/TBW (L)	0.390 (0.008)	0.393 (0.008)	−0.003	−0.005, 0.000	0.023	0.394 (0.007)	0.396 (0.006)	−0.002	−0.004, −0.001	0.011
BCM (kg)	31.84 (3.64)	31.07 (3.38)	0.78	−0.21, 1.77	0.121	23.21 (2.40)	23.12 (2.92)	0.09	−0.61, 0.79	0.798
VFA (cm^2^)	86.76 (29.51)	92.05 (32.31)	−5.29	−13.78, 3.20	0.221	118.12 (35.81)	116.71 (45.01)	1.40	−9.16, 11.96	0.794
TBW/FFM	73.96 (0.27)	74.02 (0.27)	−0.05	−0.13, 0.02	0.159	73.78 (0.24)	73.85 (0.25)	−0.07	−0.13, 0.00	0.047
**Impedance**										
5 kHz-RA Z (Ω)	325.45 (33.89)	325.12 (34.21)	0.33	−9.13, 9.79	0.945	396.10 (38.19)	393.86 (40.63)	2.24	−8.37, 12.85	0.678
5 kHz-LA Z (Ω)	326.49 (32.25)	327.05 (35.33)	−0.56	−9.85, 8.72	0.905	395.90 (37.69)	391.27 (39.64)	4.63	−5.80, 15.07	0.383
5 kHz-TR Z (Ω)	29.58 (2.87)	29.35 (2.91)	0.23	−0.57, 1.03	0.573	31.16 (3.41)	30.82 (3.29)	0.34	−0.58, 1.26	0.466
5 kHz-RL Z (Ω)	240.22 (27.12)	236.35 (31.55)	3.87	−4.13, 11.87	0.341	275.62 (35.21)	266.54 (32.65)	9.08	−0.32, 18.48	0.058
5 kHz-LL Z (Ω)	243.64 (29.43)	239.96 (33.83)	3.68	−4.96, 12.32	0.402	276.78 (35.30)	268.19 (31.90)	8.58	−0.78, 17.94	0.072
50 kHz-RA Z (Ω)	289.06 (31.71)	290.45 (32.47)	−1.39	−10.28, 7.51	0.759	359.23 (35.52)	357.63 (36.70)	1.60	−8.18, 11.38	0.748
50 kHz-LA Z (Ω)	291.75 (30.17)	293.94 (33.61)	−2.19	−10.93, 6.55	0.622	360.91 (35.54)	357.21 (36.56)	3.70	−6.07, 13.47	0.456
50 kHz-TR Z (Ω)	26.19 (2.66)	26.06 (2.67)	0.12	−0.62, 0.86	0.743	27.90 (3.10)	27.65 (3.21)	0.25	−0.60, 1.10	0.563
50 kHz-RL Z (Ω)	213.60 (24.69)	211.60 (28.50)	1.99	−5.27, 9.25	0.589	249.24 (31.38)	242.57 (29.08)	6.67	−1.70, 15.05	0.118
50 kHz-LL Z (Ω)	216.77 (26.85)	215.04 (30.48)	1.73	−6.11, 9.58	0.663	250.57 (31.52)	243.82 (28.61)	6.75	−1.62, 15.12	0.113
250 kHz-RA Z (Ω)	259.89 (29.83)	262.00 (30.22)	−2.11	−10.45, 6.22	0.618	326.35 (33.14)	324.88 (33.55)	1.48	−7.59, 10.54	0.749
250 kHz-LA Z (Ω)	262.90 (28.28)	266.15 (31.25)	−3.26	−11.42, 4.91	0.433	328.87 (33.40)	325.66 (33.61)	3.21	−5.91, 12.33	0.489
250 kHz-TR Z (Ω)	23.06 (2.52)	23.06 (2.51)	0.00	−0.70, 0.70	0.996	24.91 (2.89)	24.68 (3.05)	0.24	−0.56, 1.04	0.561
250 kHz-RL Z (Ω)	193.42 (23.03)	192.17 (26.61)	1.26	−5.52, 8.03	0.715	226.93 (28.57)	221.79 (26.59)	5.14	−2.50, 12.77	0.186
250 kHz-LL Z (Ω)	196.76 (25.20)	195.98 (28.36)	0.78	−6.55, 8.11	0.835	228.49 (28.80)	223.45 (26.36)	5.04	−2.62, 12.70	0.196
**Reactance**										
5 kHz-RA Xc (Ω)	13.98 (2.34)	13.51 (2.31)	0.47	−0.18, 1.12	0.153	14.50 (2.22)	14.44 (2.89)	0.07	−0.60, 0.73	0.840
5 kHz-LA Xc (Ω)	13.46 (2.17)	12.96 (2.11)	0.50	−0.09, 1.10	0.098	14.14 (2.18)	13.72 (2.53)	0.43	−0.20, 1.05	0.179
5 kHz-TR Xc (Ω)	1.49 (0.31)	1.45 (0.33)	0.04	−0.05, 0.12	0.395	1.50 (0.32)	1.45 (0.29)	0.05	−0.04, 0.14	0.258
5 kHz-RL Xc (Ω)	9.82 (2.26)	9.14 (2.22)	0.68	0.05, 1.31	0.033	9.82 (2.22)	8.90 (2.03)	0.92	0.33, 1.51	0.002
5 kHz-LL Xc (Ω)	9.92 (2.35)	9.18 (2.26)	0.74	0.09, 1.38	0.025	9.52 (2.08)	8.97 (1.98)	0.56	0.00, 1.11	0.051
50 kHz-RA Xc (Ω)	28.36 (3.34)	27.59 (3.30)	0.76	−0.16, 1.69	0.106	31.11 (3.55)	30.74 (4.32)	0.36	−0.67, 1.40	0.488
50 kHz-LA Xc (Ω)	28.17 (3.24)	27.15 (3.32)	1.02	0.11, 1.93	0.028	30.58 (3.51)	29.97 (4.03)	0.61	−0.39, 1.61	0.234
50 kHz-TR Xc (Ω)	2.58 (0.46)	2.46 (0.45)	0.11	−0.01, 0.24	0.078	2.44 (0.53)	2.38 (0.46)	0.06	−0.08, 0.20	0.424
50 kHz-RL Xc (Ω)	19.50 (3.85)	18.47 (3.73)	1.03	−0.02, 2.09	0.055	20.39 (3.98)	18.82 (3.93)	1.58	0.50, 2.66	0.004
50 kHz-LL Xc (Ω)	19.58 (4.04)	18.45 (3.97)	1.13	0.02, 2.25	0.046	20.29 (4.11)	18.79 (3.57)	1.50	0.42, 2.58	0.007
250 kHz-RA Xc (Ω)	27.08 (3.07)	26.89 (3.74)	0.18	−0.74, 1.11	0.698	33.06 (3.89)	32.61 (4.10)	0.45	−0.62, 1.53	0.407
250 kHz-LA Xc (Ω)	29.34 (3.28)	28.77 (4.08)	0.58	−0.42, 1.57	0.256	35.47 (4.10)	34.90 (4.27)	0.56	−0.57, 1.70	0.327
250 kHz-TR Xc (Ω)	1.60 (0.47)	1.48 (0.48)	0.11	−0.02, 0.25	0.085	1.46 (0.52)	1.42 (0.53)	0.03	−0.11, 0.18	0.633
250 kHz-RL Xc (Ω)	12.12 (2.34)	11.73 (2.30)	0.39	−0.26, 1.03	0.238	13.42 (2.72)	12.82 (2.54)	0.60	−0.13, 1.33	0.107
250 kHz-LL Xc (Ω)	12.28 (2.38)	11.78 (2.39)	0.50	−0.16, 1.17	0.137	13.27 (2.71)	12.59 (2.31)	0.68	−0.02, 1.39	0.058
**Phase angle**										
5 kHz-RA PhA (°)	2.47 (0.41)	2.39 (0.42)	0.08	−0.03, 0.20	0.167	2.10 (0.28)	2.10 (0.36)	0.00	−0.08, 0.09	0.931
5 kHz-LA PhA (°)	2.37 (0.37)	2.28 (0.37)	0.09	−0.01, 0.19	0.089	2.05 (0.29)	2.01 (0.30)	0.04	−0.04, 0.13	0.275
5 kHz-TR PhA (°)	2.89 (0.56)	2.83 (0.55)	0.07	−0.09, 0.22	0.393	2.75 (0.53)	2.71 (0.59)	0.04	−0.11, 0.19	0.587
5 kHz-RL PhA (°)	2.34 (0.49)	2.21 (0.46)	0.13	0.00, 0.26	0.051	2.04 (0.38)	1.91 (0.35)	0.13	0.03, 0.23	0.015
5 kHz-LL PhA (°)	2.34 (0.49)	2.20 (0.48)	0.14	0.01, 0.28	0.041	1.97 (0.35)	1.92 (0.36)	0.05	−0.04, 0.15	0.269
50 kHz-RA PhA (°)	5.66 (0.64)	5.48 (0.57)	0.18	0.01, 0.36	0.036	4.98 (0.47)	4.94 (0.54)	0.04	−0.09, 0.18	0.513
50 kHz-LA PhA (°)	5.56 (0.59)	5.32 (0.56)	0.24	0.08, 0.40	0.003	4.88 (0.49)	4.82 (0.49)	0.06	−0.07, 0.19	0.387
50 kHz-TR PhA (°)	5.69 (1.08)	5.46 (1.07)	0.23	−0.07, 0.53	0.127	5.03 (1.07)	4.99 (1.11)	0.04	−0.26, 0.33	0.800
50 kHz-RL PhA (°)	5.26 (0.95)	5.02 (0.89)	0.24	−0.02, 0.50	0.069	4.69 (0.71)	4.45 (0.73)	0.24	0.05, 0.44	0.014
50 kHz-LL PhA (°)	5.21 (0.99)	4.93 (0.90)	0.28	0.01, 0.55	0.041	4.64 (0.76)	4.42 (0.68)	0.22	0.02, 0.42	0.032
250 kHz-RA PhA (°)	6.00 (0.51)	5.89 (0.45)	0.11	−0.03, 0.24	0.117	5.82 (0.40)	5.76 (0.47)	0.05	−0.06, 0.17	0.361
250 kHz-LA PhA (°)	6.43 (0.50)	6.21 (0.52)	0.22	0.08, 0.36	0.003	6.20 (0.49)	6.16 (0.49)	0.04	−0.09, 0.18	0.539
250 kHz-TR PhA (°)	4.00 (1.24)	3.71 (1.18)	0.30	−0.04, 0.64	0.085	3.36 (1.14)	3.29 (1.16)	0.07	−0.24, 0.38	0.662
250 kHz-RL PhA (°)	3.60 (0.61)	3.52 (0.66)	0.08	−0.09, 0.26	0.340	3.39 (0.57)	3.32 (0.51)	0.07	−0.08, 0.22	0.333
250 kHz-LL PhA (°)	3.60 (0.65)	3.46 (0.63)	0.14	−0.04, 0.32	0.122	3.34 (0.61)	3.24 (0.52)	0.10	−0.06, 0.26	0.238
50 kHz-WB PhA (°)	5.49 (0.71)	5.29 (0.61)	0.20	0.01, 0.39	0.036	4.87 (0.49)	4.75 (0.52)	0.12	−0.02, 0.25	0.086

^1^Mean (SD). ^2^Two Sample *t*-test; Two sample test for equality of proportions. NC, normal control; MCI, mild cognitive impairment; D, mean difference; CI, confidence interval; ICW, intra-cellular water; ECW, extra-cellular water; TBW, total body water; SLM, soft lean mass; FFM, fat free mass; SMM, skeletal muscle mass; PBF, percent body fat; WHR, waist to hip ratio; BCM, body cell mass; VFA, visceral fat area; RA, right arm; LA, left arm; TR trunk; RL, right leg; LL, left leg; Z, impedance; Xc, reactance; PhA, phase angle; WB, whole body.

### 3.2 Prediction model

**Figure 1:**
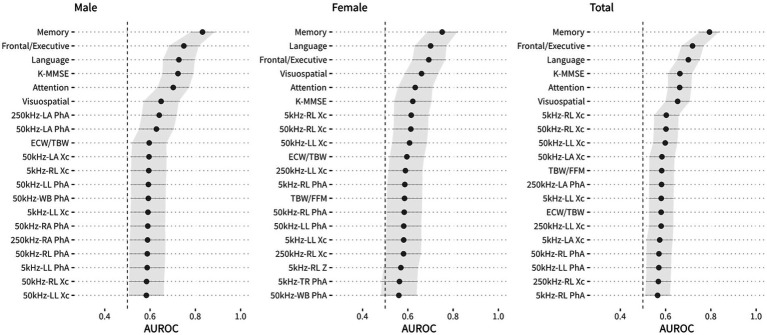
The area under the receiver operating curve (AUROC) for each variable, including variables of the neuropsychological screening tools and BIA, and DeLong’s 95% confidence intervals for AUROC values.

AUROC values were used to compare the classification accuracies of individual variables from neuropsychological screening tools and BIA measurements for the diagnosis of MCI, as shown in Figure 1. The AUROC values of the scores on the SNSB-II memory, frontal, language, and attention domains and the K-MMSE in the male group were more than 0.7, and the maximum AUROC value was obtained with the SNSB-II memory score (0.832). In the female group, AUROC values for SNSB II memory and language were more than 0.7, and the maximum value was obtained with the SNSB-II memory score (0.752), similar to the male group. In the classification model for both sexes, AUROC values of more than 0.7 were obtained for the SNSB-II memory, frontal, and language scores, and the maximum AUROC value was 0.794 (SNSB-II memory score). The AUROC values of K-MMSE scores in males, females, and both sexes were 0.723, 0.622, and 0.663, respectively. According to previous studies, the AUROC values of MMSE scores used for screening for MCI were approximately 0.69–0.78 (Ahn et al., 2010; Shim et al., 2015; Lee et al., 2018). The AUROC value of classification accuracy using the MMSE score from our data was 0.663, showing similar accuracy to that of previous reports.

**Figure 2:**
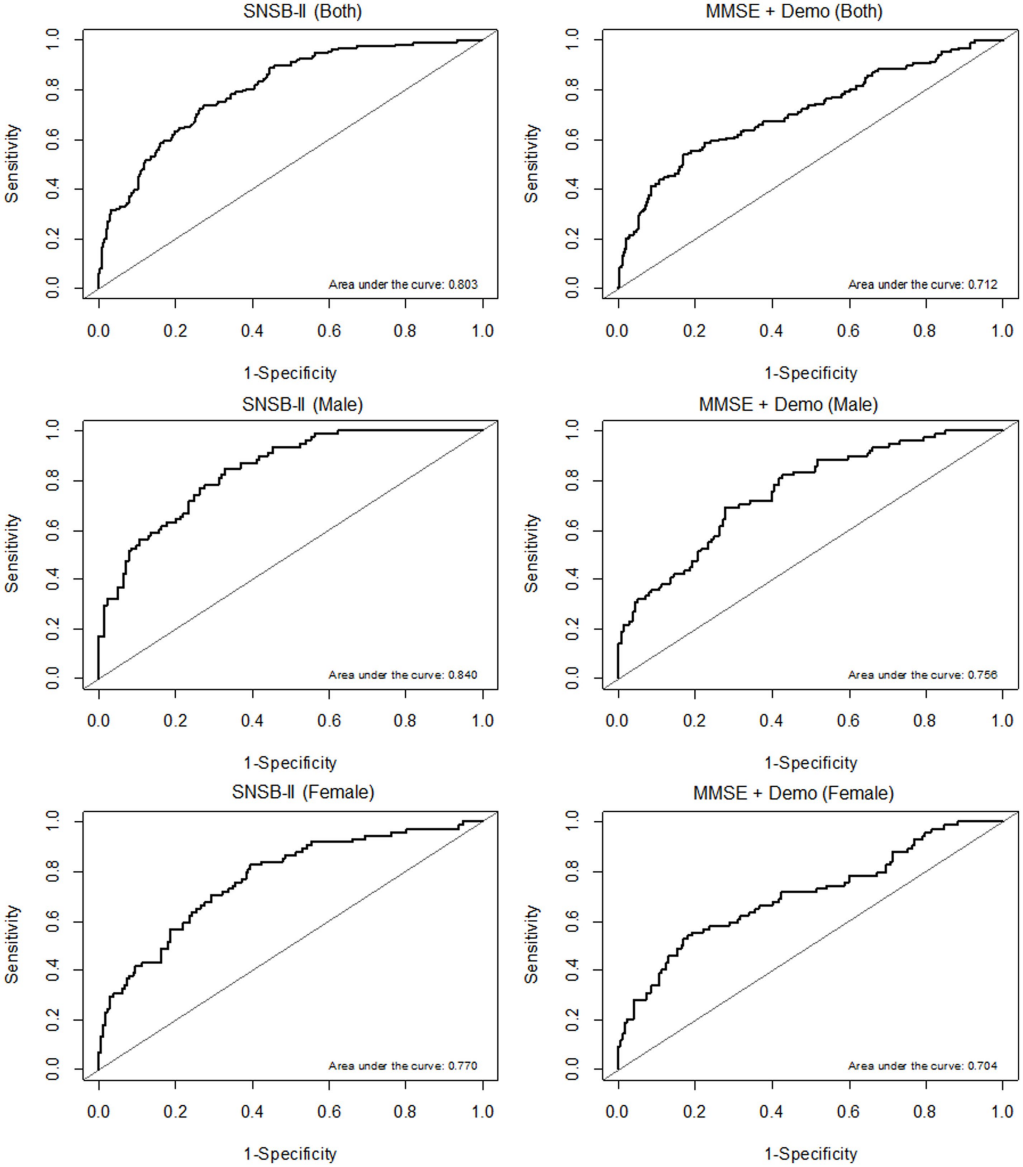
ROC curves of prediction models according to sex (both sexes, males, and females, from top to bottom). **(A)** AUROC results of the prediction models including the 5 domain scores of the SNSB-II. **(B)** AUROC results of the prediction models including K-MMSE scores and demographic information.

A prediction model for multiple regression analysis was constructed with the 5 domain scores of SNSB-II (attention, language, memory, visuospatial, and frontal/executive function), and the AUROC values were calculated to evaluate the accuracy of MCI diagnosis. The AUROC values of the prediction model including the 5 domain scores of the SNSB-II were 0.803 in both sexes, 0.840 in the male group, and 0.770 in the female group. In addition, the accuracy of MCI diagnosis was calculated with the AUROC values of the prediction model including the K-MMSE total score and demographic information as variables. The AUROC values of the prediction model including the K-MMSE scores and demographic information were 0.712 in both sexes, 0.756 in the male group, and 0.704 in the female group. The ROC curves of prediction models for MCI diagnosis including SNSB-II and K-MMSE scores are shown in Figure 2.

**Figure 3:**
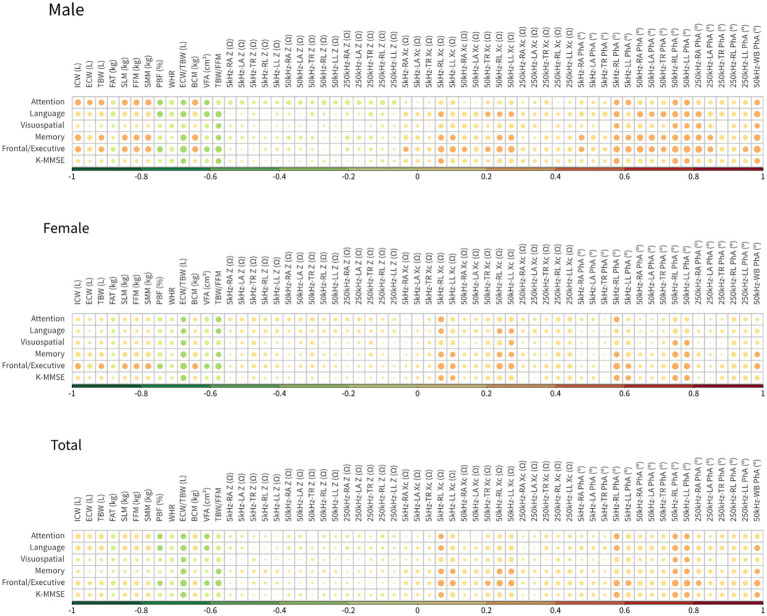
Heatmap of Pearson’s correlation coefficients between neuropsychological examination tools and BIA measures.

The correlations between BIA variables and neuropsychological test scores are shown in a heatmap in Figure 3. The highest correlation coefficient was between extra-cellular water/total body water (ECW/TBW) (BIA variable) and age; this value was 0.449. The correlation coefficient of total body water/fat free mass (TBW/FFM) (BIA variable) and years of education was −0.190, that of the reactance of the right leg at 5 kHz (BIA variable) and SNSB-II attention scores was 0.268, that of TBW/FFM (BIA variable) and SNSB-II language scores was −0.226, that of the phase angle of the left leg at 50 kHz (BIA variable) and SNSB-II visuospatial scores was 0.238, that of ECW/TBW (BIA variable) and SNSB-II memory scores was −0.270, that of the phase angle of the right leg at 50 kHz (BIA variable) and SNSB-II frontal scores was 0.336, and that of ECW/TBW (BIA variable) and K-MMSE scores was −0.254. Previous studies have reported that the correlation coefficients between some of the variables of resting-state EEG data and K-MMSE scores were in the range of 0.3–0.6 (Choi et al., 2019; Doan et al., 2021). The present correlation analyses show that the BIA variables reflected both body composition and characteristic differences in cognitive decline, as indicated by neuropsychological test scores. Therefore, it is expected that the accuracy of MCI prediction can be increased by combining BIA variables and K-MMSE scores.

**Figure 4:**
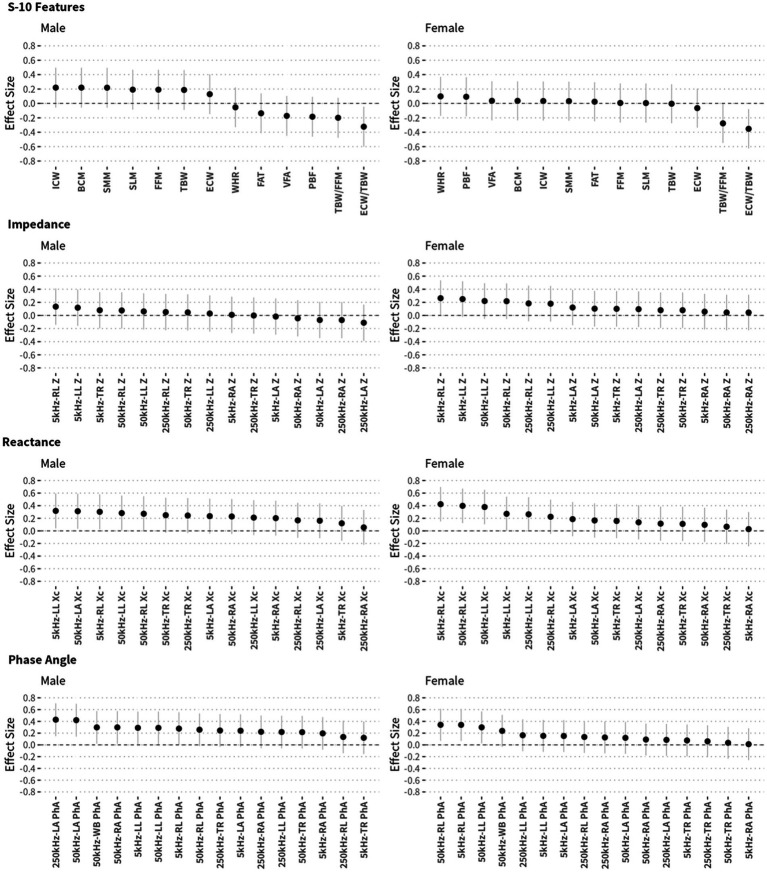
Effect sizes of each BIA measurement and their 95% confidence intervals according to sex. Effect sizes (Cohen’s *d*) were calculated to compare NC and MCI participants.

**Figure 5:**
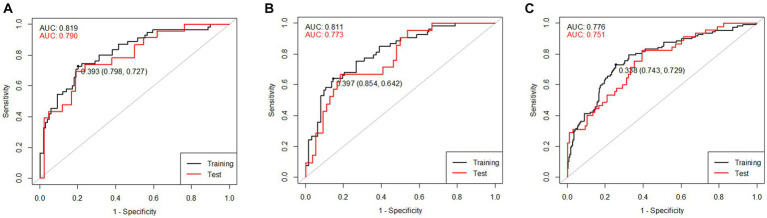
ROC curves of the training and test sets of the combined models including K-MMSE scores and BIA variables: **(A)** combined model for males, **(B)** combined model for females, and **(C)** combined model for both sexes.

The effect size of the difference between NCs and MCI was also analyzed to select BIA variables to include in the prediction model, as shown in Figure 4. There was a difference in the effect size according to sex; the effect size was 0.4 or more in the phase angles in the male group and in the reactance in the female group. There were also many variables with an effect size of 0.2 or more. Based on the correlation and effect size results, prediction models were developed to distinguish between NCs and individuals with MCI. The developed prediction models included the K-MMSE score and BIA variables; these combined models were compared (according to AUROC values) with prediction models including SNSB-II scores only. Three combined models were constructed: one each for males, females, and both sexes. This was because BIA variables showed significant differences between males and females and to enable comparison. The multiple regression equation of the combined model for males is as follows:


(1) $$\begin{array}{l} \mathrm{MCIprediction}\_M=0.090\times Edu–0.656\times \mathrm{MMSE}–\\ 0.084\times FFM+204.640\times \mathrm{ECWR}–1.568\times 50\;\mathrm{kHz}\_\\ LA\_Xc+1.489\times 250\;\mathrm{kHz}\_LA\_Xc+7.149\times \\ 50\;\mathrm{kHz}\_LA\_PhA–7.430\times 250\;\mathrm{kHz}\_LA\_PhA+\\ 3.528\times 50\;\mathrm{kHz}\_WB\_PhA-71.174\text{,} \end{array}$$

where Edu is years of education, MMSE is the K-MMSE total score, FFM is fat-free mass, ECWR is the ratio of extracellular water to total body water (ECW/TBW), 50 kHz_LA_Xc is the reactance of the left arm at 50 kHz, 250 kHz_LA_Xc is the reactance of the left arm at 250 kHz frequency, 50 kHz_LA_PhA is the phase angle of the left arm at 50 kHz, 250kHz_LA_PhA is the phase angle of the left arm at 250 kHz, and 50kHz_WB_PhA is the phase angle of the whole body at 50 kHz. To validate the developed prediction model, the AUROC values were calculated by dividing all male participants into a training set (of 154 participants; 70%) and a test set (of 65 participants; 30%). The AUROC values of the training and test sets for the male combined model, MCIprediction_M, were 0.819 and 0.790, respectively, and the ROC curve and AUROC values are shown in Figure 5A. The multiple regression equation of the combined model for females is as follows:


(2) $$\begin{array}{l} \mathrm{MCIprediction}\_F=0.153\times Edu–0.329\times \mathrm{MMSE}+\\ 0.349\times \mathrm{wt}+7.460\times TBW–0.370\times PBF–534.768\times \\ \mathrm{ECWRTR}–369.779\times \mathrm{ECWRLL}–9.657\times BCM–\\ 1.172\times 50\;\mathrm{kHz}\_LL\_Z+1.230\times 250\;\mathrm{kHz}\_\\ LL\_Z–2.357\times 5\;\mathrm{kHz}\_RL\_PhA+398.151\text{,} \end{array}$$

where wt is weight, TBW is total body water, PBF is percent body fat, ECWRTR is the ratio of extracellular water to segmental water in the trunk [extra-cellular water of trunk (ECWTR)/segmental water of trunk (SWTR)], ECWRLL is the ratio of extracellular water to segmental water in the left leg [ECWLL/segmental water of the left leg (SWLL)], BCM is body cell mass, 50kHz_LL_Z is the impedance of the left leg at 50 kHz, 250 kHz_LL_Z is the impedance of the left leg at 250 kHz, and 5kHz_RL_PhA is the phase angle of the right leg at 5 kHz. The AUROC values were calculated by dividing all female participants into a training set (of 176 participants; 70%) and a test set (of 75 participants; 30%). The AUROC values of the training and test sets of the female combined model, MCIprediction_F, were 0.811 and 0.773, respectively, and the ROC curve and AUROC values are shown in Figure 5B. The multiple regression equation of the combined model for both sexes is as follows.


(3) $$\begin{array}{l} \mathrm{MCIprediction}\_B=\mathrm{sex}\times \\ \left(\begin{array}{l} -70.869–0.351\times \mathrm{wt}–0.266\times \mathrm{MMSE}+\\ 1.084\times ICW+0.395\times PBF+155.479\times \\ \mathrm{ECWR}+0.321\times 50\;\mathrm{kHz}\_RL\_Xc \end{array}\right)+\\ 0.132\times Edu–0.339\times \mathrm{MMSE}+0.351\times \mathrm{wt}–1.136\times \\ ICW–0.333\times PBF–65.770\times \mathrm{ECWR}–130.934\times \\ \mathrm{ECWRLA}+0.180\times \mathrm{5kHz}\_RA\_Xc-0.153\times \\ 50\mathrm{kHz}\_RL\_Xc+0.698\times 250\;\mathrm{kHz}\_RL\_PhA–9.001 \end{array}$$

where male is 1 and female is 0 and ICW is intracellular water. The AUROC values were calculated by dividing all participants into a training set (of 329 participants; 70%) and a test set (of 141 participants; 30%). The AUROC values of the training and test sets of the combined model, MCIprediction_B, were 0.776 and 0.751, respectively, and the ROC curve and AUROC values are shown in Figure 5C.

The combined model of MMSE and BIA variables for MCI screening exhibited an AUROC value in Figure 5, comparable to that of the comprehensive neuropsychological battery. While slight variations were observed based on dataset sizes or the extent of separation between training and test sets, it was evident that this model possesses sufficient potential for effective screening MCI. Given the current trend of 10–20% MCI incidence (Langa and Levine, 2014) and the rapid aging of populations, there’s an increasing need for prompt and cost-effective MCI screening technologies. This study’s results showed the feasibility of utilizing the developed models.

Next, to develop an improved MCI screening technique that combined K-MMSE scores and BIA variables, we further employed various machine learning algorithms beyond regression approach. The prediction models generated by these algorithms were compared according to the obtained AUROC values. The training data for machine learning consisted of SNSB-II and K-MMSE scores, BIA variables, and their combinations. ELASTIC-NET, svmRBF, RF, and XGBOOST algorithms were utilized for MCI screening.

**Figure 6:**
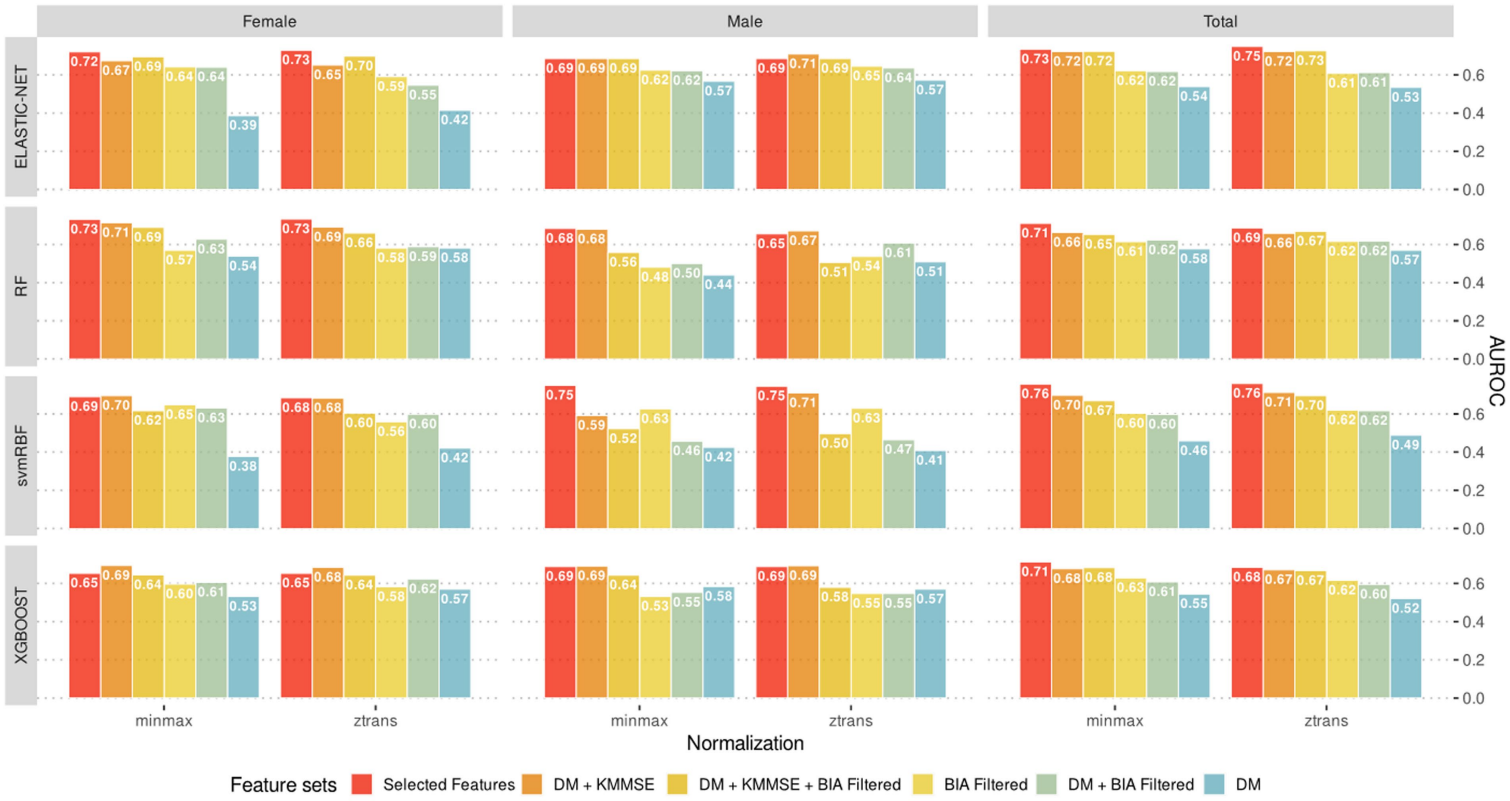
The best predictive models (evaluated by test sets) according to the combination of sex-stratified datasets, feature sets, normalization methods and machine learning algorithms.

Figure 6 presents the classification models developed with machine learning algorithms and their corresponding AUROC values. Among these prediction models, the svmRBF algorithm achieved the highest AUROC value of 0.76; the AUROC values of the RF algorithm for females and the svmRBF algorithm for males were 0.73 and 0.75, respectively. The AUROC values of the other machine learning models ranged from 0.65 to 0.73, with no significant difference from those of the svmRBF model and RF algorithm. Notably, the AUROC values of the prediction models developed with machine learning algorithms did not exhibit significant improvements from the subset regression models developed. This could be attributed to the limited amount of data available for the analysis, which might not have been sufficient for robust machine learning analysis.

Expanding the sample size will likely lead to more accurate and reliable prediction models using machine learning techniques. Further studies with more diverse datasets including various ethnics, cultural background and body fat mass will improve clinical utility of the machine learning models for MCI prediction.

## 4 Discussion

With the rapid aging of the population, there’s a growing need for cognitive impairment detection tests among the elderly. However, while simple tools like MMSE or MoCA are less reliable for MCI diagnosis, comprehensive neuropsychological batteries like CERAD and SNSB-II, commonly used for diagnosis, present drawbacks: lengthy administration (around 1.5 h), costly, and need specialized experts. A rapid and accurate MCI screening method enables the prompt and precise identification of individuals at risk of MCI. Directing those identified to in-depth hospital examinations for precise diagnoses saves time and resources compared to extensive testing for more general population group. Therefore, there’s a demand for technology that enables relatively accurate, rapid, and cost-effective MCI screening. Diagnosing mild Cognitive Impairment (MCI) requires a comprehensive approach involving various criteria (Langa and Levine, 2014). These criteria serve as guidelines for clinicians in making a comprehensive judgment regarding the diagnosis of MCI, particularly when considering various potential underlying causes and clinical presentations. Certainly, diagnosing Mild Cognitive Impairment (MCI) relies on an expert’s assessment, combining multiple indicators. Relying on a single indicator for diagnosis is challenging. Having a reliable and simple screening tool for identifying individuals who require detailed MCI diagnosis would significantly aid in the diagnostic process.

Recently, several papers have reported an association between MCI or dementia due to AD and BIA measures. These studies have shown a significant association (ranging from 0.2 to 0.4, value of *p* <0.05) of BIA variables with MCI or dementia due to AD (Bae et al., 2017; Fujisawa et al., 2017; Mathys et al., 2017). However, the specific BIA variables that vary are inconsistent across papers. Furthermore, the underlying physiological mechanism explaining why BIA variables differ between MCI, AD, and normal controls has not been clearly elucidated. Therefore, further studies are needed to investigate these mechanisms. In summary, there is still a need for reliable and efficient MCI screening techniques given population aging. Although BIA variables have shown promising associations with MCI and AD, more comprehensive research is needed to identify the specific BIA variables indicative of cognitive impairment and to better understand the underlying physiological mechanisms.

The MMSE has been widely utilized in clinical practice as an effective and sensitive test for detecting and screening cognitive impairment and dementia (Benson et al., 2005; Arevalo-Rodriguez et al., 2015). It has a high accuracy of 92% in detecting dementia, with sensitivity ranging from 78 to 84% and specificity ranging from 87 to 91% (Tsoi et al., 2015). However, the MMSE does have some limitations; it can be influenced by factors such as the socio-educational backgrounds of participants and practice, and it has low sensitivity for the early stage of cognitive decline (Scazufca et al., 2009; Duff et al., 2012; Carnero-Pardo, 2014). By integrating the MMSE with BIA variables, which can be easily and quickly performed, it is possible to provide a more convenient and accessible means to detect cognitive impairment in its early stage and recommend for MCI screening. This combination has the potential to overcome some of the limitations associated with use of the MMSE alone, providing a more comprehensive and reliable assessment of cognitive status especially in the early stage.

The comparison of BIA variables among individuals with MCI or dementia is a relatively recent research topic. While there may be minimal disparities in muscle mass between individuals with cognitive impairment and normal cognition, differences in nutritional status or water ratios appear in the reactance or phase angles of segmental BIA measurements. Based on these observations, we constructed a prediction model for MCI that combined BIA variables with MMSE scores. The aim of incorporating BIA variables into the prediction model alongside MMSE scores was to identify new factors for detecting cognitive impairment. The study showed some BIA variables related to muscle mass, extra/intra-cellular water ratio and lower level of bioimpedance features such as reactance and phase angle as risk factors of cognitive impairment. Through the development and evaluation of the prediction model, we aimed to provide evidence of the feasibility of using BIA as a screening tool.

The screening accuracy of the MMSE for cognitive impairment varies with ethnic, language, and demographic factors. To address this variability, compensation tables with MMSE cut-off scores for MCI and dementia screening have been utilized accounting for differences in sex, age, and education level. On the other hand, BIA variables are influenced by factors such as sex, height, weight, age, and ethnicity. Previous studies demonstrated differences in some BIA variables for MCI even after adjusting for age, sex, height, and weight (Doan et al., 2022a). However, there is currently no standardized approach for predicting dementia or MCI using BIA variables. This study used the results of previous studies as a reference to develop an MCI screening model. It is the first to propose combining MMSE scores with BIA variables to enhance the model’s predictive power for detecting MCI. Future studies aiming at developing refined prediction models by accounting for various demographic factors can facilitate more accurate and reliable screening tools for dementia and MCI. Healthcare professionals would effectively screen individuals with diverse backgrounds.

It’s reported that BIA variables serve as valuable indicators for monitoring and screening various diseases and conditions. However, it remains unclear how much these disease-dependent BIA variables would work as confounding factors in MCI screening. Previous studies have shown differences in BIA variables between individuals with diabetes and those without, particularly in PhA (Jun et al., 2018). Considering the previous research findings (Doan et al., 2022a,b, 2023), differences in BIA variables between MCI and normal groups persist even after adjusting for conditions like hyperlipidemia, diabetes, and CNS disorders. In our study, there was a difference in diabetes prevalence between the MCI and NC groups in the male group, but no significant difference was observed in the female group. Nevertheless, when looking at the model’s AUC values, the difference between the male and female groups was not substantial. This indicates that the model’s ability to screen for MCI remains consistent regardless of diabetes status.

In this study, we investigated combinations of machine learning algorithms, BIA variables, and K-MMSE total scores for screening for MCI. However, we did not observe a significant improvement in diagnostic accuracy with machine learning algorithms compared to more classical regression models. Importantly, the small size of the dataset used in this study may have influenced the results. With a larger dataset, machine learning models may be able to capture more patterns and relationships, leading to improved diagnostic accuracy. Additionally, including individuals with a wider range of demographic factors and considering other relevant variables may also contribute to better results in future research.

### 4.1 Limitations

One limitation of this study is the application of a matched-samples design, which may restrict the generalizability of the findings. The sample used in this study may not be representative of the entire Korean population, further limiting the external validity of the results. Additionally, as BIA variables can vary based on geographical region, participant race, age, sex, BMI, and other factors, it is essential to develop a prediction model for MCI screening based on data from more diverse populations, considering a wider range of regions, age, sex, BMI values, and other factors. This will ensure the broader applicability of a screening tool to predict mild cognitive impairment.

While it is widely acknowledged that MoCA is more effective in detecting MCI than MMSE, our cohort center, the GARD Cohort Research Center, has been collecting data including MMSE, Seoul Neuropsychological Screening Battery (SNSB) II, and BIA since 2017. MoCA is not widely used in Korea yet and it is one of our study limitations.

## 5 Conclusion

The estimated prevalence of mild cognitive impairment (MCI) in those over 65 is approximately 10 to 20%. As comprehensive testing for all suspected MCI cases is challenging, a simple and accurate screening tool is needed. To examine the possibility of screening for cognitive impairment in elderly people, we developed a prediction model for mild cognitive impairment (MCI) based on the multivariate regressing model by combining multifrequency bioimpedance analysis (MF-BIA) variables and total scores of the Mini-Mental State Examination (MMSE). Pearson correlation analysis was performed to examine the associations between MF-BIA variables and scores on the MMSE and Seoul Neuropsychological Screening Battery Scores II (SNSB II). Weak correlations were observed with the highest value of 0.336.

To assess the model performance, the AUROC values of the combined model of MF-BIA and MMSE were compared with that of the model using the SNSB-II scores, which were popularly used diagnostic tool for cognitive impairment in South Korea, by dividing the dataset into training and test sets (70 and 30%, respectively). The AUROC values of the SNSB-II were 0.803 in both sexes, 0.840 in the male group, and 0.770 in the female group. The AUROC values of the training and test sets of the combined model were 0.776 and 0.751 in both sexes, 0.819 and 0.790 in the male group, and 0.811 and 0.773 in the female group, respectively. The maximum AUROC value of the model developed with machine learning was 0.76 (for the svmRBF algorithm), which did not significantly differ from the combined model.

The accuracy reduction of the combined model was not significant compared to that of the model based on a standard diagnostic battery (SNSB-II). Accounting for the ease of use, short measurement time, and the low cost to implement in clinics, the BIA and MMSE combined model is considered to have sufficient potential for screening for MCI.

## Data availability statement

The original contributions presented in the study are included in the article/Supplementary material, further inquiries can be directed to the corresponding author.

## Ethics statement

The studies involving humans were approved by The Institutional Review Board (IRB) of Chonnam National University Hospital and Chosun University Hospital (IRB number: CNUH-2019-279, CHOSUN 2016-10-005-017). The studies were conducted in accordance with the local legislation and institutional requirements. The participants provided their written informed consent to participate in this study.

## Author contributions

M-HJ: Conceptualization, Formal analysis, Investigation, Methodology, Writing – original draft, Writing – review & editing. BK: Formal analysis, Writing – review & editing. KK: Data curation, Writing – review & editing. KL: Data curation, Writing – review & editing. JK: Funding acquisition, Project administration, Writing – original draft, Writing – review & editing.
