# Supplementary material for: A screening method for mild cognitive impairment in elderly individuals combining bioimpedance and MMSE
Source: Front Aging Neurosci. 2024 Jan 24;16:1307204. doi: 10.3389/fnagi.2024.1307204 (PMC10847325; doi:10.3389/fnagi.2024.1307204)
Supplement: Supplementary file 1 [file Table_1.DOCX]

Supplementary Material

# Supplementary Tables

Table A1. MCI classification accuracy of uni-variable by AUROC value

|  | | **Male** | |  | **Female** | |  | **Total** | |
| --- | --- | --- | --- | --- | --- | --- | --- | --- | --- |
|  |  | **AUROC^1^** | **95% CI^2^** |  | **AUROC^1^** | **95% CI^2^** |  | **AUROC^1^** | **95% CI^2^** |
| ***SNSB-II & K-MMSE*** | | | | | |  |  |  |  |
|  | Attention | 0.702 | 0.632, 0.772 |  | 0.633 | 0.557, 0.709 |  | 0.662 | 0.610, 0.714 |
|  | Language | 0.727 | 0.657, 0.798 |  | 0.701 | 0.630, 0.771 |  | 0.700 | 0.650, 0.751 |
|  | Visuospatial | 0.649 | 0.571, 0.726 |  | 0.660 | 0.587, 0.734 |  | 0.653 | 0.600, 0.707 |
|  | Memory | 0.832 | 0.778, 0.885 |  | 0.752 | 0.685, 0.818 |  | 0.794 | 0.751, 0.836 |
|  | Frontal/Executive | 0.749 | 0.683, 0.815 |  | 0.692 | 0.620, 0.765 |  | 0.719 | 0.670, 0.768 |
|  | K-MMSE | 0.723 | 0.655, 0.792 |  | 0.622 | 0.539, 0.704 |  | 0.663 | 0.610, 0.717 |
| ***S-10 Features*** | | | | | |  |  |  |  |
|  | ICW (L) | 0.564 | 0.485, 0.644 |  | 0.507 | 0.423, 0.590 |  | 0.521 | 0.465, 0.576 |
|  | ECW (L) | 0.541 | 0.460, 0.621 |  | 0.533 | 0.449, 0.617 |  | 0.535 | 0.479, 0.590 |
|  | TBW (L) | 0.555 | 0.475, 0.635 |  | 0.515 | 0.431, 0.599 |  | 0.526 | 0.470, 0.581 |
|  | FAT (kg) | 0.530 | 0.448, 0.613 |  | 0.545 | 0.460, 0.629 |  | 0.516 | 0.457, 0.574 |
|  | SLM (kg) | 0.557 | 0.476, 0.637 |  | 0.513 | 0.429, 0.597 |  | 0.525 | 0.469, 0.580 |
|  | FFM (kg) | 0.555 | 0.475, 0.635 |  | 0.514 | 0.429, 0.598 |  | 0.525 | 0.469, 0.581 |
|  | SMM (kg) | 0.564 | 0.485, 0.644 |  | 0.508 | 0.424, 0.592 |  | 0.521 | 0.466, 0.577 |
|  | PBF (%) | 0.550 | 0.470, 0.631 |  | 0.529 | 0.448, 0.610 |  | 0.522 | 0.467, 0.578 |
|  | WHR | 0.515 | 0.433, 0.598 |  | 0.542 | 0.456, 0.629 |  | 0.512 | 0.453, 0.571 |
|  | ECW/TBW (L) | 0.596 | 0.520, 0.672 |  | 0.596 | 0.520, 0.672 |  | 0.580 | 0.527, 0.634 |
|  | BCM (kg) | 0.564 | 0.485, 0.644 |  | 0.506 | 0.422, 0.590 |  | 0.521 | 0.465, 0.576 |
|  | VFA (cm²) | 0.539 | 0.458, 0.621 |  | 0.526 | 0.443, 0.610 |  | 0.516 | 0.458, 0.573 |
|  | TBW/FFM | 0.556 | 0.477, 0.636 |  | 0.586 | 0.508, 0.663 |  | 0.583 | 0.528, 0.637 |
| ***Impedance*** | | | | | |  |  |  |  |
|  | 5kHz-RA Z (Ω) | 0.511 | 0.430, 0.591 |  | 0.520 | 0.439, 0.601 |  | 0.539 | 0.483, 0.595 |
|  | 5kHz-LA Z (Ω) | 0.508 | 0.428, 0.589 |  | 0.466 | 0.386, 0.545 |  | 0.541 | 0.485, 0.597 |
|  | 5kHz-TR Z (Ω) | 0.528 | 0.448, 0.609 |  | 0.529 | 0.452, 0.607 |  | 0.535 | 0.480, 0.590 |
|  | 5kHz-RL Z (Ω) | 0.533 | 0.452, 0.615 |  | 0.569 | 0.493, 0.646 |  | 0.561 | 0.506, 0.616 |
|  | 5kHz-LL Z (Ω) | 0.526 | 0.445, 0.607 |  | 0.560 | 0.483, 0.637 |  | 0.555 | 0.499, 0.610 |
|  | 50kHz-RA Z (Ω) | 0.496 | 0.415, 0.577 |  | 0.488 | 0.408, 0.569 |  | 0.531 | 0.475, 0.586 |
|  | 50kHz-LA Z (Ω) | 0.505 | 0.424, 0.585 |  | 0.529 | 0.450, 0.608 |  | 0.534 | 0.479, 0.590 |
|  | 50kHz-TR Z (Ω) | 0.520 | 0.440, 0.600 |  | 0.522 | 0.444, 0.600 |  | 0.529 | 0.473, 0.585 |
|  | 50kHz-RL Z (Ω) | 0.521 | 0.440, 0.602 |  | 0.552 | 0.476, 0.629 |  | 0.550 | 0.494, 0.605 |
|  | 50kHz-LL Z (Ω) | 0.509 | 0.428, 0.589 |  | 0.550 | 0.472, 0.627 |  | 0.545 | 0.489, 0.600 |
|  | 250kHz-RA Z (Ω) | 0.486 | 0.405, 0.567 |  | 0.489 | 0.409, 0.569 |  | 0.527 | 0.472, 0.583 |
|  | 250kHz-LA Z (Ω) | 0.519 | 0.438, 0.599 |  | 0.527 | 0.448, 0.606 |  | 0.530 | 0.474, 0.586 |
|  | 250kHz-TR Z (Ω) | 0.508 | 0.427, 0.588 |  | 0.520 | 0.441, 0.599 |  | 0.524 | 0.468, 0.579 |
|  | 250kHz-RL Z (Ω) | 0.515 | 0.434, 0.596 |  | 0.537 | 0.460, 0.614 |  | 0.541 | 0.486, 0.597 |
|  | 250kHz-LL Z (Ω) | 0.503 | 0.423, 0.584 |  | 0.464 | 0.387, 0.542 |  | 0.538 | 0.482, 0.594 |
| ***Reactance*** | | | | | |  |  |  |  |
|  | 5kHz-RA Xc (Ω) | 0.553 | 0.473, 0.632 |  | 0.487 | 0.402, 0.572 |  | 0.539 | 0.481, 0.597 |
|  | 5kHz-LA Xc (Ω) | 0.578 | 0.498, 0.658 |  | 0.555 | 0.470, 0.640 |  | 0.574 | 0.516, 0.632 |
|  | 5kHz-TR Xc (Ω) | 0.547 | 0.468, 0.626 |  | 0.560 | 0.482, 0.637 |  | 0.553 | 0.498, 0.608 |
|  | 5kHz-RL Xc (Ω) | 0.594 | 0.516, 0.672 |  | 0.615 | 0.539, 0.691 |  | 0.603 | 0.549, 0.657 |
|  | 5kHz-LL Xc (Ω) | 0.591 | 0.514, 0.668 |  | 0.582 | 0.503, 0.660 |  | 0.581 | 0.526, 0.635 |
|  | 50kHz-RA Xc (Ω) | 0.573 | 0.495, 0.651 |  | 0.523 | 0.439, 0.606 |  | 0.564 | 0.507, 0.621 |
|  | 50kHz-LA Xc (Ω) | 0.595 | 0.518, 0.673 |  | 0.553 | 0.471, 0.634 |  | 0.584 | 0.528, 0.640 |
|  | 50kHz-TR Xc (Ω) | 0.569 | 0.489, 0.648 |  | 0.539 | 0.464, 0.614 |  | 0.549 | 0.494, 0.603 |
|  | 50kHz-RL Xc (Ω) | 0.585 | 0.507, 0.662 |  | 0.613 | 0.537, 0.690 |  | 0.602 | 0.548, 0.656 |
|  | 50kHz-LL Xc (Ω) | 0.584 | 0.506, 0.661 |  | 0.607 | 0.531, 0.684 |  | 0.598 | 0.545, 0.652 |
|  | 250kHz-RA Xc (Ω) | 0.541 | 0.460, 0.622 |  | 0.535 | 0.455, 0.616 |  | 0.550 | 0.493, 0.607 |
|  | 250kHz-LA Xc (Ω) | 0.564 | 0.484, 0.645 |  | 0.543 | 0.464, 0.623 |  | 0.562 | 0.505, 0.618 |
|  | 250kHz-TR Xc (Ω) | 0.559 | 0.479, 0.640 |  | 0.528 | 0.451, 0.605 |  | 0.539 | 0.483, 0.594 |
|  | 250kHz-RL Xc (Ω) | 0.539 | 0.460, 0.618 |  | 0.581 | 0.505, 0.658 |  | 0.568 | 0.514, 0.622 |
|  | 250kHz-LL Xc (Ω) | 0.562 | 0.484, 0.641 |  | 0.589 | 0.514, 0.665 |  | 0.580 | 0.526, 0.634 |
| ***Phase Angle*** | | | | | |  |  |  |  |
|  | 5kHz-RA PhA (º) | 0.551 | 0.472, 0.630 |  | 0.510 | 0.429, 0.592 |  | 0.505 | 0.449, 0.562 |
|  | 5kHz-LA PhA (º) | 0.572 | 0.493, 0.651 |  | 0.541 | 0.460, 0.621 |  | 0.532 | 0.476, 0.588 |
|  | 5kHz-TR PhA (º) | 0.544 | 0.466, 0.622 |  | 0.563 | 0.484, 0.641 |  | 0.543 | 0.488, 0.598 |
|  | 5kHz-RL PhA (º) | 0.583 | 0.504, 0.661 |  | 0.587 | 0.509, 0.664 |  | 0.565 | 0.510, 0.619 |
|  | 5kHz-LL PhA (º) | 0.587 | 0.509, 0.665 |  | 0.555 | 0.477, 0.633 |  | 0.548 | 0.492, 0.603 |
|  | 50kHz-RA PhA (º) | 0.590 | 0.513, 0.667 |  | 0.530 | 0.449, 0.610 |  | 0.525 | 0.469, 0.580 |
|  | 50kHz-LA PhA (º) | 0.628 | 0.551, 0.705 |  | 0.542 | 0.462, 0.621 |  | 0.550 | 0.495, 0.605 |
|  | 50kHz-TR PhA (º) | 0.579 | 0.499, 0.658 |  | 0.524 | 0.447, 0.601 |  | 0.531 | 0.476, 0.587 |
|  | 50kHz-RL PhA (º) | 0.588 | 0.508, 0.667 |  | 0.584 | 0.506, 0.662 |  | 0.571 | 0.515, 0.626 |
|  | 50kHz-LL PhA (º) | 0.593 | 0.515, 0.670 |  | 0.582 | 0.505, 0.659 |  | 0.570 | 0.516, 0.625 |
|  | 250kHz-RA PhA (º) | 0.589 | 0.511, 0.667 |  | 0.545 | 0.462, 0.628 |  | 0.556 | 0.499, 0.612 |
|  | 250kHz-LA PhA (º) | 0.640 | 0.563, 0.718 |  | 0.540 | 0.462, 0.617 |  | 0.582 | 0.527, 0.637 |
|  | 250kHz-TR PhA (º) | 0.557 | 0.477, 0.637 |  | 0.531 | 0.454, 0.608 |  | 0.534 | 0.479, 0.590 |
|  | 250kHz-RL PhA (º) | 0.545 | 0.464, 0.625 |  | 0.543 | 0.466, 0.620 |  | 0.537 | 0.482, 0.593 |
|  | 250kHz-LL PhA (º) | 0.568 | 0.488, 0.647 |  | 0.550 | 0.473, 0.627 |  | 0.549 | 0.494, 0.605 |
|  | 50kHz-WB PhA (º) | 0.592 | 0.516, 0.669 |  | 0.560 | 0.482, 0.639 |  | 0.543 | 0.488, 0.598 |
| ^1^Area under the ROC curve | | | | | | | | | |
| ^2^DeLong's 95% confidence interval for AUROC | | | | | | | | | |

Table A2. Correlation coefficients between the questionnaire, SNSB-II domains and K-MMSE, and demography information and bioimpedance data

| **Variables** | **SNSB-II**  **Attention** | **SNSB-II**  **Language** | **SNSB-II**  **Visuospatial** | **SNSB-II**  **Memory** | **SNSB-II**  **Frontal** | **K-MMSE** |
| --- | --- | --- | --- | --- | --- | --- |
| K-MMSE | 0.470 | 0.481 | 0.366 | 0.552 | 0.594 | 1 |
| AGE (yrs.) | -0.340 | -0.295 | -0.221 | -0.393 | -0.453 | -0.272 |
| Education (yrs.) | 0.482 | 0.472 | 0.377 | 0.461 | 0.628 | 0.511 |
| HEIGHT (cm) | 0.193 | 0.119 | 0.145 | 0.228 | 0.287 | 0.078 |
| WEIGHT (kg) | -0.051 | -0.049 | -0.040 | 0.028 | 0.009 | 0.015 |
| BMI (kg/m^2) | -0.150 | -0.110 | -0.108 | -0.087 | -0.137 | -0.017 |
| ICW (l) | 0.121 | 0.079 | 0.116 | 0.194 | 0.255 | 0.115 |
| ECW (l) | 0.075 | 0.028 | 0.060 | 0.132 | 0.182 | 0.059 |
| TBW (l) | 0.104 | 0.060 | 0.095 | 0.172 | 0.229 | 0.094 |
| FAT (kg) | -0.135 | -0.108 | -0.105 | -0.083 | -0.132 | -0.047 |
| SLM (kg) | 0.108 | 0.065 | 0.100 | 0.176 | 0.235 | 0.099 |
| FFM (kg) | 0.111 | 0.067 | 0.103 | 0.179 | 0.239 | 0.100 |
| SMM (kg) | 0.121 | 0.078 | 0.116 | 0.193 | 0.254 | 0.115 |
| PBF (%) | -0.173 | -0.123 | -0.137 | -0.145 | -0.222 | -0.085 |
| WHR | -0.111 | -0.089 | -0.115 | -0.057 | -0.101 | -0.063 |
| ECW/TBW | -0.205 | -0.215 | -0.222 | -0.270 | -0.324 | -0.254 |
| BCM (kg) | 0.120 | 0.080 | 0.115 | 0.193 | 0.255 | 0.116 |
| VFA (cm^2) | -0.178 | -0.144 | -0.148 | -0.135 | -0.208 | -0.099 |
| TBW/FFM | -0.236 | -0.226 | -0.196 | -0.204 | -0.293 | -0.191 |
| 5kHz.RA.Z (Ω) | 0.106 | 0.054 | 0.019 | 0.028 | 0.038 | -0.018 |
| 5kHz.LA.Z (Ω) | 0.121 | 0.099 | 0.058 | 0.051 | 0.076 | 0.014 |
| 5kHz.TR.Z (Ω) | 0.152 | 0.082 | 0.146 | 0.152 | 0.128 | 0.070 |
| 5kHz.RL.Z (Ω) | 0.106 | 0.113 | 0.046 | 0.086 | 0.102 | 0.039 |
| 5kHz.LL.Z (Ω) | 0.124 | 0.110 | 0.050 | 0.067 | 0.097 | 0.044 |
| 50kHz.RA.Z (Ω) | 0.106 | 0.056 | 0.020 | 0.019 | 0.024 | -0.033 |
| 50kHz.LA.Z (Ω) | 0.118 | 0.093 | 0.059 | 0.042 | 0.060 | 0.000 |
| 50kHz.TR.Z (Ω) | 0.136 | 0.076 | 0.126 | 0.124 | 0.010 | 0.045 |
| 50kHz.RL.Z (Ω) | 0.074 | 0.085 | 0.015 | 0.050 | 0.052 | 0.002 |
| 50kHz.LL.Z (Ω) | 0.102 | 0.088 | 0.018 | 0.035 | 0.058 | 0.011 |
| 250kHz.RA.Z (Ω) | 0.106 | 0.055 | 0.021 | 0.014 | 0.018 | -0.032 |
| 250kHz.LA.Z (Ω) | 0.118 | 0.090 | 0.060 | 0.036 | 0.055 | -0.002 |
| 250kHz.TR.Z (Ω) | 0.110 | 0.065 | 0.118 | 0.105 | 0.073 | 0.017 |
| 250kHz.RL.Z (Ω) | 0.055 | 0.063 | -0.010 | 0.025 | 0.020 | -0.022 |
| 250kHz.LL.Z (Ω) | 0.083 | 0.065 | -0.005 | 0.008 | 0.028 | -0.013 |
| 5kHz.RA.Xc (Ω) | -0.005 | 0.016 | -0.006 | 0.032 | 0.048 | 0.043 |
| 5kHz.LA.Xc (Ω) | 0.006 | 0.051 | 0.027 | 0.044 | 0.089 | 0.080 |
| 5kHz.TR.Xc (Ω) | 0.073 | 0.030 | 0.077 | 0.093 | 0.078 | 0.057 |
| 5kHz.RL.Xc (Ω) | 0.268 | 0.213 | 0.180 | 0.237 | 0.314 | 0.223 |
| 5kHz.LL.Xc (Ω) | 0.170 | 0.170 | 0.170 | 0.208 | 0.241 | 0.203 |
| 50kHz.RA.Xc (Ω) | 0.058 | 0.077 | 0.006 | 0.096 | 0.093 | 0.028 |
| 50kHz.LA.Xc (Ω) | 0.069 | 0.108 | 0.031 | 0.095 | 0.120 | 0.062 |
| 50kHz.TR.Xc (Ω) | 0.125 | 0.092 | 0.144 | 0.165 | 0.193 | 0.140 |
| 50kHz.RL.Xc (Ω) | 0.195 | 0.213 | 0.188 | 0.230 | 0.291 | 0.195 |
| 50kHz.LL.Xc (Ω) | 0.199 | 0.206 | 0.202 | 0.216 | 0.265 | 0.198 |
| 250kHz.RA.Xc (Ω) | 0.087 | 0.083 | 0.027 | 0.089 | 0.074 | -0.006 |
| 250kHz.LA.Xc (Ω) | 0.085 | 0.124 | 0.060 | 0.069 | 0.079 | 0.006 |
| 250kHz.TR.Xc (Ω) | 0.061 | 0.102 | 0.027 | 0.037 | 0.123 | 0.102 |
| 250kHz.RL.Xc (Ω) | 0.159 | 0.119 | 0.136 | 0.173 | 0.168 | 0.102 |
| 250kHz.LL.Xc (Ω) | 0.179 | 0.143 | 0.093 | 0.163 | 0.166 | 0.151 |
| 5kHz.RA.PhA (°) | -0.082 | -0.018 | -0.025 | 0.019 | 0.030 | 0.059 |
| 5kHz.LA.PhA (°) | -0.076 | -0.018 | -0.010 | 0.010 | 0.051 | 0.077 |
| 5kHz.TR.PhA (°) | -0.009 | -0.012 | -0.027 | -0.009 | 0.002 | 0.004 |
| 5kHz.RL.PhA (°) | 0.246 | 0.169 | 0.177 | 0.231 | 0.321 | 0.245 |
| 5kHz.LL.PhA (°) | 0.104 | 0.115 | 0.156 | 0.199 | 0.224 | 0.208 |
| 50kHz.RA.PhA (°) | -0.036 | 0.035 | -0.011 | 0.106 | 0.093 | 0.065 |
| 50kHz.LA.PhA (°) | -0.040 | 0.027 | -0.020 | 0.073 | 0.085 | 0.071 |
| 50kHz.TR.PhA (°) | 0.063 | 0.042 | 0.076 | 0.100 | 0.141 | 0.117 |
| 50kHz.RL.PhA (°) | 0.180 | 0.198 | 0.227 | 0.262 | 0.336 | 0.251 |
| 50kHz.LL.PhA (°) | 0.158 | 0.176 | 0.238 | 0.242 | 0.286 | 0.233 |
| 250kHz.RA.PhA (°) | -0.005 | 0.055 | 0.016 | 0.127 | 0.102 | 0.027 |
| 250kHz.LA.PhA (°) | -0.034 | 0.055 | 0.004 | 0.052 | 0.044 | 0.009 |
| 250kHz.TR.PhA (°) | 0.037 | 0.096 | -0.007 | 0.011 | 0.120 | 0.110 |
| 250kHz.RL.PhA (°) | 0.144 | 0.092 | 0.170 | 0.199 | 0.191 | 0.143 |
| 250kHz.LL.PhA (°) | 0.127 | 0.098 | 0.114 | 0.179 | 0.160 | 0.170 |
| 50kHz.WB.PhA (°) | 0.086 | 0.139 | 0.123 | 0.212 | 0.254 | 0.182 |
